# Effects of aerobic exercise on cardiac function and gene expression of NADPH oxidases in diaphragm muscle of rats with aortic stenosis-induced heart failure

**DOI:** 10.3389/fphys.2023.1182303

**Published:** 2023-06-08

**Authors:** Mariana Janini Gomes, Ana Karenina Sabela, Isabele Tiburcio Pecin Ferreira, Sérgio Luiz Borges de Souza, Gustavo Augusto Ferreira Mota, Vitor Loureiro da Silva, Dijon Henrique Salomé de Campos, Aline Regina Ruiz Lima, Marianna Rabelo Carvalho, Silmeia Garcia Zanati Bazan, Camila Renata Corrêa, Antônio Carlos Cicogna, Marina Politi Okoshi, Francis Lopes Pacagnelli

**Affiliations:** ^1^ Department of Kinesiology and Sport Management, Texas A&M University, College Station, TX, United States; ^2^ Physiotherapy Department, UNOESTE, Presidente Prudente, Brazil; ^3^ Post-graduate Program, Animal Science, UNOESTE, Presidente Prudente, Brazil; ^4^ Botucatu Medical School, Sao Paulo State University (UNESP), Botucatu, Brazil; ^5^ Federal University of Mato Grosso Do Sul (UFMS), Campo Grande, Brazil; ^6^ Pathology Department, Sao Paulo State University (UNESP), Botucatu, Brazil

**Keywords:** aortic stenosis, heart failure, diaphragm, oxidative stress, aerobic physical exercise

## Abstract

We evaluated the influence of aerobic physical exercise (EX) on gene-encoding proteins associated with oxidative stress in diaphragm muscle of rats with aortic stenosis-induced heart failure (HF). Wistar male rats were divided into four groups: Control sedentary (C); Control exercise (C-Ex); Sedentary aortic stenosis (AS); Aortic stenosis exercise (AS-Ex). Exercised rats trained 5 times a week for 10 weeks on a treadmill. Statistical analysis was performed by ANOVA or Kruskal–Wallis test. In the final echocardiogram, animals with aortic stenosis subjected to exercise demonstrated improvement in systolic function compared to the sedentary aortic stenosis group. In diaphragm muscle, the activity of antioxidant enzymes, malondialdehyde malondialdehyde concentration, protein carbonylation, and protein expression of p65 and its inhibitor IκB did not differ between groups. Alterations in gene expression of sources that generate reactive species of oxygen were observed in AS-Ex group, which showed decreased mRNA abundance of NOX2 and NOX4 compared to the aortic stenosis group (*p* < 0.05). We concluded that aerobic exercise has a positive impact during heart failure, ameliorating systolic dysfunction and biomarkers of oxidative stress in diaphragm muscle of rats with aortic stenosis-induced heart failure.

## 1 Introduction

Heart failure (HF) affects 6.5 million adults in the U.S. and this number is expected to increase to more than 8 million by 2030. It accounts for about 8.5% of all heart diseases deaths in the country and it is a leading cause of mortality globally (Heidenreich et al., 2022). Exercise intolerance, the hallmark symptom of HF, directly affects the quality of life of patients and is associated with poor prognosis and increased mortality (Del Buono et al., 2019). This symptom has a multifactorial nature, which includes intrinsic abnormalities that occur in both limb and inspiratory muscles.

The diaphragm is the main inspiratory muscle, and diaphragm dysfunction exacerbates the disease pathophysiology and morbidity. Several molecular mechanisms involved in diaphragm dysfunction associated with HF have been described in both experimental and clinical studies, which include impaired muscle contractility, impaired Ca^2+^ handling, increased inflammation, oxidative stress, and muscle fiber atrophy and weakness (Salah et al., 2022).

Oxidative stress, defined as an imbalance between the production of reactive oxygen species (ROS) and antioxidant capacity, has been implicated in respiratory muscle dysfunction during HF (Van Hees et al., 2008; Van Hees et al., 2010; Jaenisch et al., 2018). Furthermore, NADPH oxidase, which is one of the major sources of ROS in skeletal muscle, has been associated with deterioration of diaphragm contractile function (Bechara et al., 2014; Ahn et al., 2015; Ahn et al., 2017; Reyes et al., 2019). The abnormal production of ROS in skeletal muscle initiate activation of the transcription factor NF-kappa B (NF-𝜅B), which was previously reported to be activated in response to increased oxidative stress in skeletal muscle of rats with heart failure (Martinez et al., 2016).

To date, there is no specific pharmacological therapy to treat or prevent diaphragm myopathy. Regular exercise is the most effective known treatment. Clinical and experimental studies have demonstrated several positive effects of aerobic exercise on skeletal muscle, including an increase in antioxidant capacity and reduction in oxidative stress in the setting of heart failure (de Souza et al., 2015; Gomes et al., 2016; Mangner et al., 2016; Jaenisch et al., 2018). Despite all information regarding the protective effects of exercise counteracting diaphragm muscle abnormalities, our current knowledge of the mechanisms by which exercise improves skeletal muscle health is insufficient. We hypothesized that the modulation of NADPH oxidases underlies the beneficial effects of aerobic exercise in the prevention or attenuation of diaphragm myopathy associated with HF. A deeper understanding of the effects of aerobic exercise and the role of oxidative stress on diaphragm muscle is still lacking to inform the development of effective therapeutic approaches for diaphragm myopathy associated with HF.

Ascending aortic stenosis in rats is a useful model to study chronic pressure overload-induced cardiac remodeling and diaphragm abnormalities. In this model, 3- 4-week-old rats are subjected to a clip being placed around the ascending aorta. After clip placement, aorta diameter is preserved; as rats grow, stenosis progressively develops. The model has the advantage that, despite rapid left ventricular hypertrophy onset, ventricular dysfunction and heart failure occur slowly (Gomes et al., 2017), similar to what is seen in humans. In this study we evaluated the influence of aerobic exercise on exercise tolerance, cardiac parameters, and biomarkers of oxidative stress in the diaphragm muscle of rats with aortic stenosis-induced heart failure.

## 2 Material and methods

This study was registered and approved by Sao Paulo State University - UNESP, Botucatu, under protocol nº: 1138–2015. The experiments were performed in accordance with *Guide for the Care and Use of Laboratory Animals* (*National Research Council)* and followed the recommendations of ARRIVE (Kilkenny et al., 2010; The National Academies Collection, 2011).

Three-to four-week-old male Wistar rats, purchased from the Central Animal House, Botucatu Medical School, Sao Paulo State University, Brazil, weighing ∼70–90 g, were anesthetized with ketamine hydrochloride (60 mg/kg/ip Dopalen^®^, Sespo, Jacareí, São Paulo, Brazil) and xylazine hydrochloride (10 mg/kg/ip Anasedan^®^, Sespo, Jacareí, São Paulo, Brazil) and aortic stenosis was induced by placing a 0.6 mm stainless-steel clip on the ascending aorta via a thoracic incision (Gomes et al., 2017). During the surgery, animals were manually ventilated by positive pressure with 100% oxygen. Sham operated rats were used as controls. All surgeries were performed by the same researcher, specialized in this procedure. At the end of the surgery, rats received subcutaneous isotonic solution (2 mL/kg, saline solution^®^, ADV Farma, Nova Odessa, São Paulo, Brazil) and were placed on a heated surface until recovery from anesthesia, they were, then, orally medicated with Metamizol Sodium^®^ (30 mg/kg), Biovet, Vargem Grande Paulista, São Paulo, Brazil).

Animal care during the postoperative period, 5 days followed the surgery, consisted of daily cleaning cages, antibiotic and analgesic administration, and surgical wound antisepsis with Chlorhexidine Diglyconate 1% (10 mg/mL, Riohex 1%^®^, Sao Jose do Rio Preto, Sao Paulo, Brazil). Animals were housed in collective polypropylene boxes with chromed wire covers, padded with sterile Pinus shavings (4 animals per box), under identical conditions on an inverted cycle (12-12 h light/dark). The animal facility staff provided water and food *ad libitum* to the animals during the experimental period.

The animals were initially randomized into two groups: Control (C = 16) and Aortic Stenosis (AS = 16). At 18 weeks after surgery, both groups (C and AS) were randomly redistributed in either sedentary (C, n = 8; and AS, n = 8) or aerobic exercise groups (C-Ex, n = 8; and AS-Ex, n = 8). The exercise protocol is described in detail below.

### 2.1 Maximal exercise test and aerobic exercise training

**FIGURE 1:**
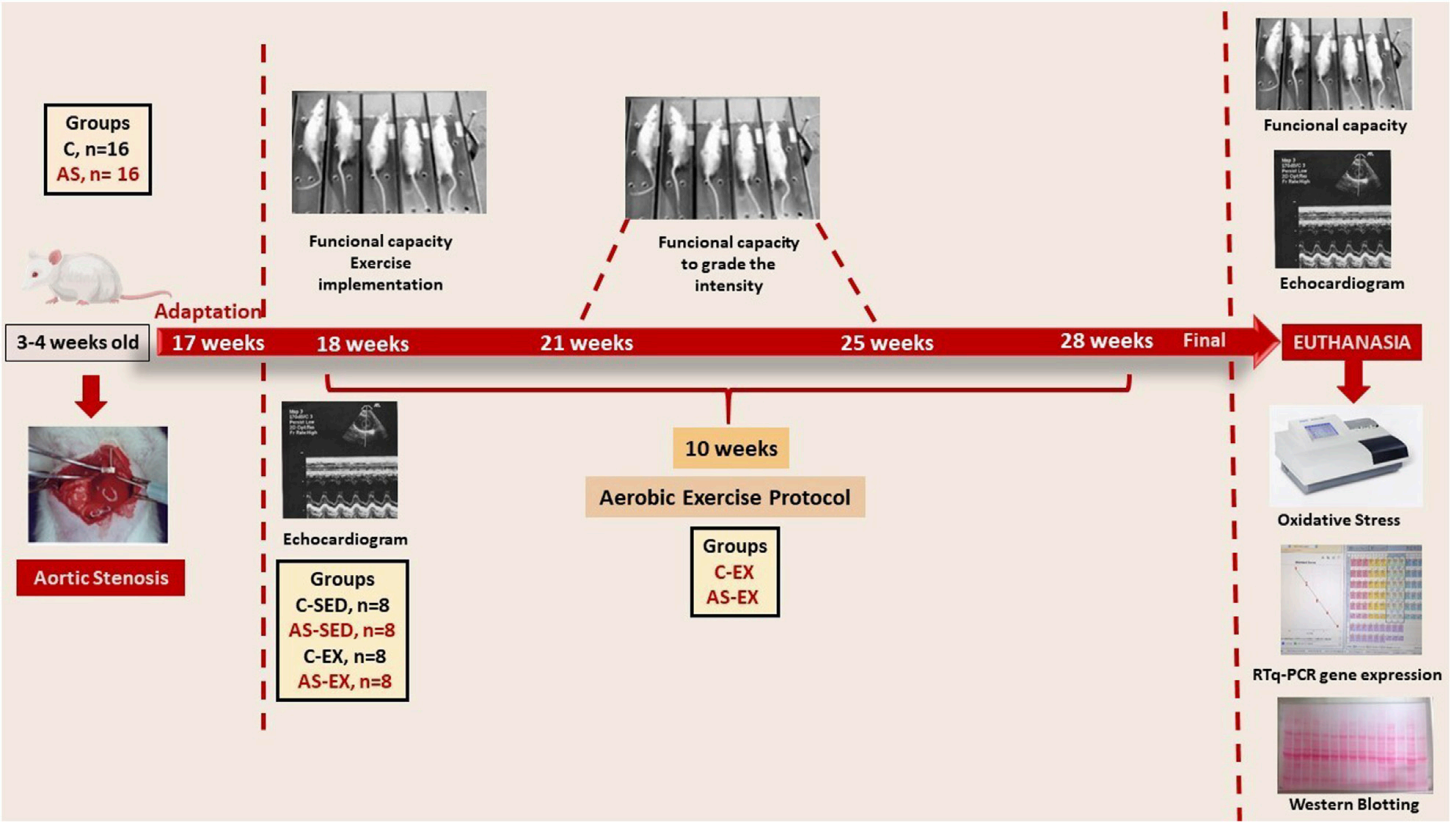
Schematic figure summarizing the experimental design.

Aerobic exercise program was performed on a treadmill for rats (Insight Instruments-Brazil). There was an adaptation period, where animals ran at 5 m/min for 7 days. Subsequently, all animals underwent a maximal exercise test before and after exercise protocol. Additionally, exercised groups were re-evaluated in weeks 3 and 7 to adjust exercise intensity. Each rat was tested individually on a treadmill according to a previously described protocol (Pacagnelli et al., 2016), until exhaustion, which was determined when rats refused to run even after electric stimulation or were unable to coordinate steps. Maximum speed was recorded and total distance calculated. (Figure 1).

Exercised groups (C-Ex and AS-Ex) underwent an aerobic exercise program, which consisted of running on a treadmill, 5x/week, for 10 weeks. Exercise duration was gradually increased from weeks 1–6 (10–20 min). From the sixth week on, each session consisted of 20 min of running at 50% of the maximum speed attained in the maximal exercise test.

### 2.2 Cardiac evaluation by echocardiogram

Cardiac structures and left ventricular function were evaluated by transthoracic echocardiography in 18- and 28-week-old rats. The examinator was blinded to the sedentary vs. exercised groups. For examination, rats were anesthetized with ketamine hydrochloride (50 mg/kg/ip) and xylazine hydrochloride (10 mg/kg/ip) and placed in left lateral decubitus position. An echocardiograph, model Vivid S6 (General Electric Medical Systems, Tirat Carmel, Israel), equipped with a 12 MHz electronic transducer was used. Examination was performed using General Electric Medical Systems equipment (Vivid S6, Tirat Carmel-Israel), equipped with 5–11.5 MHz multifrequency transducer. Mitral and aortic transvalvular flow were evaluated with a 5.0 MHz transducer.

Morphological and functional variables of the heart were obtained (Gomes et al., 2017). Cardiac structures were measured manually, according to the American Society of Echocardiography and measured in at least five consecutive cardiac cycles. The following variables were evaluated: left ventricular (LV) diastolic diameter (LVDD); LA left atrial diameter; LV posterior wall thickness (PWT); relative wall thickness (RWT); LV mass (LVM). The relative thickness of the LV posterior wall was calculated as 2xPWDT/LVDD. The remodeling index = relative wall thickness/ventricular mass index was calculated; LV systolic function was assessed through the following indexes: MFS% midwall fractional shortening; calculated LV myocardial performance index, Tei index; TDI S′ tissue Doppler imaging (TDI) of mitral annulus systolic velocity. LV diastolic function was evaluated by the indexes: peak ratio initial filling flow velocity (E wave) and atrial contraction (A wave) of transmitral flow (Pagan et al., 2015). Additionally, was evaluated the tissue Doppler imaging (TDI) of early (TDI-E’), and late (TDI-A’) diastolic velocity of the mitral annulus (arithmetic average of the lateral and septal walls) and E/TDI-E’ ratio. The heart rate (HR) was also evaluated (Pagan et al., 2015).

### 2.3 Anatomical parameters and collection of cardiac and skeletal muscles

The day after final echocardiogram, animals were weighed, anesthetized with intraperitoneal injection of sodium pentobarbital (50 mg/kg) and euthanized by decapitation. During euthanasia, we determined the presence or absence of clinical and pathologic heart failure features. The clinical finding suggestive of heart failure was tachypnea/labored respiration. Pathologic assessment of heart failure included pleural effusion, hepatic congestion, atrial thrombi, and ascites. The left and right portions of the costal diaphragm were isolated and immediately frozen in liquid nitrogen and stored at −80 C. Left ventricle weight with inter-ventricular septum included (LV) and right ventricle weight (RV) normalized to tibia length (LV/tibia and RV/tibia respectively) were used as ventricular hypertrophy indexes (Carvalho et al., 2010).

### 2.4 Oxidative stress evaluation

Diaphragm was collected and immediately frozen in dark flasks until analysis. All tests were performed under red light so that there was no light interference.

#### 2.4.1 Antioxidants enzymes activity

Superoxide dismutase (SOD) activity was measured by inhibition of the superoxide radical reaction with pyrogallol, with absorbance values at 420 nm. Catalase was evaluated by decreasing hydrogen peroxide levels with absorbance values at 240 nm. Spectrophotometric determinations were performed using a spectrophotometer (Synergy 4, Biotek). Activity is expressed as pmol reduced H2O2/min/mg of protein. Values for the enzymatic activities were corrected by protein content. Protein was quantified in Lowry method using bovine serum albumin as standard (dos Santos et al., 2018).

#### 2.4.2 Oxidant markers

Oxidation status of diaphragm muscle was assessed by lipid peroxidation (malondialdehyde, MDA) and protein carbonylation. Muscle concentration of both oxidant markers was corrected by total protein (measured by the Bradford method).

MDA level was quantified using thiobarbituric acid (TBA) 0.67% (1:1). TBA was added to the samples, which were then heated for 45 min in a water bath at 100 ֯C. MDA was reacted with TBA in a 1:2 MDA-TBA ratio and then read at 535 nm on a Spectra Max 190 microplate reader (Molecular Devices^®^, Sunnyvale, CA, United States of America). The concentration of MDA was obtained through the molar extinction coefficient (1.56 × 10^5^ M^-1^ cm^-1^) and the absorbances of the samples and the final result was expressed in nmol/g of protein (Uchiyama and Mihara, 1978).

Protein carbonylation was measured by the nonspecific quantitative method, using 2,4-dinitrophenylhydrazine as derivatization agent (DNPH) and spectrophotometric detection. Carbonylated protein concentrations were expressed in DNPH nmol/mg protein (Samarghandian et al., 2016).

#### 2.4.3 NADPH oxidase gene expression

Gene expression of the NADPH oxidase homologs Nox2 and Nox4, and the subunits p22phox and p47phox were evaluated by RT-PCR (Martinez et al., 2010). Briefly, total RNA was extracted with TRIzol Reagent (Invitrogen Life Technologies, Carlsbad-USA) and treated with DNase I (Invitrogen Life Technologies). One microgram of RNA was reverse transcribed using a High Capacity cDNA Reverse Transcription Kit according to standard methods (Applied Biosystems, Foster City-USA). Aliquots of cDNA were then subjected to RT-PCR reaction using custom assays containing sense and antisense primers and Taqman probes (Applied Biosystems, Foster City, United States) specific to each gene: NOX2 (Rn00576710m1), NOX4 (Rn00585380m1), p22phox (Rn00577357m1), and p47phox (Rn00586945m1). Amplification and analysis were performed using Step One Plus™ PCR-RT system (Applied Biosystems, Foster City-USA). Data expression was normalized by cyclophilin (Rn00690933m1). Reactions were performed in triplicate and expression levels calculated using comparative CT method (2^−ΔΔCT^) (Lima-Leopoldo et al., 2013).

### 2.5 Protein expression

As NADPH oxidase activation is associated with activation of the NF- κB pathway, we also analyzed the protein expression of NF-κB p65 and its inhibitor IκB by Western blotting. Diaphragm muscle total protein was extracted with RIPA buffer and quantified by the Bradford method. Samples were separated on polyacrylamide gel and transferred to a nitrocellulose membrane. After blockage, membrane was incubated with primary antibody (p65 NF-κB sc-7151, phospho-p65 NF-κB (Ser 536) sc-33020, IκB-α, sc-1643 and phospho-IκB-α, sc101713; Santa Cruz Biotechnology, United States), washed with TBS and Tween, and incubated with secondary antibody conjugated to peroxidase. Super Signal® West Pico chemiluminescent substrate (Pierce Protein Research Products, Rockford, United States) was used to detect bound antibodies, which were quantified by densitometry using the software ImageJ (Gomes et al., 2016). Obtained data were normalized by GAPDH expression [GAPDH (6C5) sc-32233, Santa Cruz Biotechnology].

### 2.6 Statistical analysis

Data were expressed as mean ± deviation. Comparisons between C and AS groups was performed by Student’s t-test or Mann Whitney test. Comparisons of clinical signs were performed with Fisher’s exact test. Final comparisons of the experiment were carried out by analysis of variance (ANOVA) for a 2 × 2 factorial design followed by the Tukey test for parametric variables, which are expressed as mean ± standard deviation. Non-parametric variables were compared using the Kruskal-Wallis test followed by Dunn’s test and are expressed as median and percentiles. Data normality was evaluated by Shapiro-Wilk test (Rosa et al., 2016). Significance level considered was 5%. Systat 13.0 statistical software was used.

## 3 Results

### 3.1 Experimental groups and anatomical parameters

**FIGURE 2:**
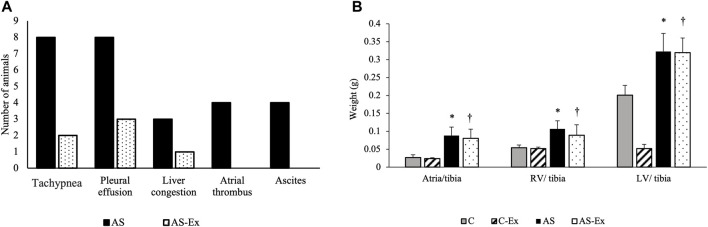
**(A)** Clinical signs of heart failure; **(B)** Anatomical parameters. Data expressed as mean ± standard deviation. Groups: C (control, n = 8); C-Ex (control physical exercise, n = 8); AS (aortic stenosis control, n = 8) AS-Ex (aortic stenosis physical exercise, n = 8). *p < 0.05 vs. C; †*p* < 0.05 vs. C-Ex. Two-way ANOVA with Tukey’s *post hoc*.

During the experimental period, two rats from C group and one rat from AS group died. In *postmortem* examination, no signs of HF were observed in animals of C and C-Ex groups. AS group presented signs of HF and AS-Ex group demonstrated a decrease in these signs (Figure 2A).

Anatomical parameters are presented in Figure 2B. LV/tibia, RV/tibia, and atria/tibia weights were higher in AS and AS-Ex groups compared to their respective controls.

### 3.2 Exercise tolerance

**FIGURE 3:**
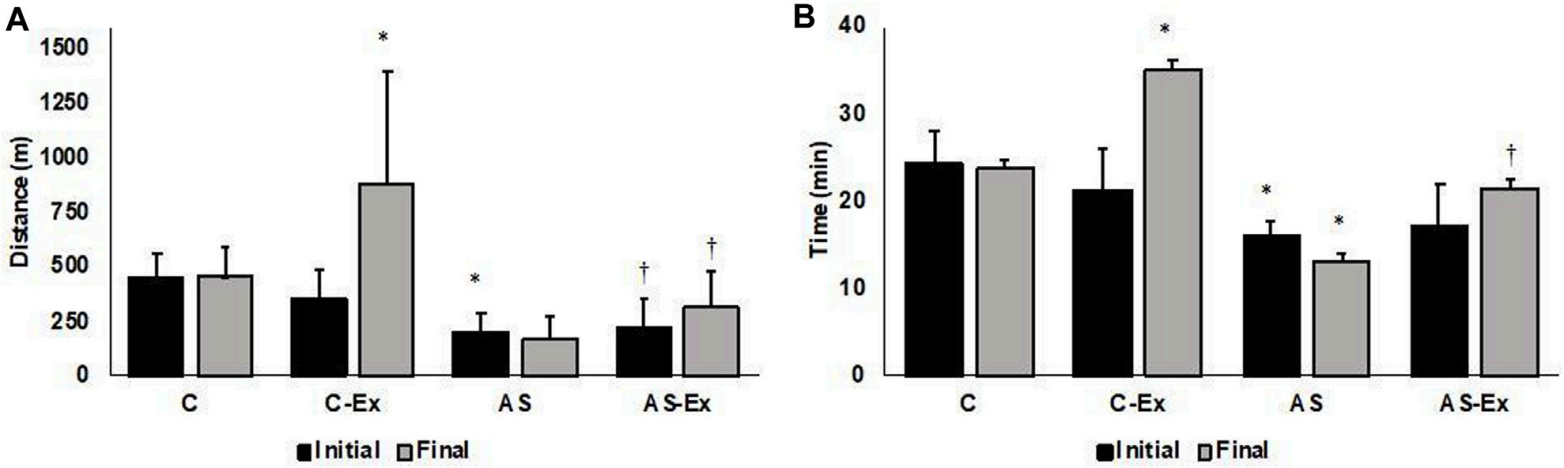
**(A)** Distance and **(B)** time achieved in the maximal exercise test. Data expressed as mean ± standard deviation. C (control, *n* = 8); C-Ex (control physical exercise, *n* = 8); AS (aortic stenosis control, *n* = 8) AS-Ex (aortic stenosis physical exercise, *n* = 8); Two-way ANOVA with Tukey’s post hoc; *p < 0.05 vs. C; ^†^ *p* < 0.05 vs. C-Ex.

Both AS and AS-Ex groups showed reduced exercise tolerance compared to their respective controls, characterized by decreased running distance and test duration (Figure 3). The average maximum speed of each group achieved in the maximum exercise test at the end of the experiment did not differ between groups (C: 18.9 ± 3.07 m/min, C-Ex: 23.5 ± 6.0 m/min, AS: 14.4 ± 8.3 m/min, AS-Ex: 14.9 ± 6.1 m/min; *p* > 0.05).

### 3.3 Structural and functional cardiac evaluation by echocardiogram

Data from the echocardiogram exam performed at 18 weeks are described in Table 1. AS group presented increased LV wall thickness, LV mass and left atrial diameter compared with control group. The remodeling index (RWT/LVM) decreased in AS group compared with control group. Functionally, AS group presented systolic dysfunction (reduced PWSS, LV, and MFS) and diastolic dysfunction (E/A) compared to control group.

**TABLE 1:** Echocardiographic structural and functional data (18 weeks).

Parameters	Groups	
	C (n = 14)	AS (n = 15)
HR (bpm)	316 ± 40.89	327 ± 41.40
LVDD/BW (mg/kg)	15.44 ± 2.01	18.10 ± 2.57†
PWT (mm)	1.65 ± 0.13	2.84 ± 0.17†
LA/BW (mg/Kg)	10.19 ± 1.32	18.48 ± 3.00†
LVM (g/kg)	0.91 ± 0.21	2.24 ± 0.40†
RWT (mm)	0.45 ± 0.03	0.76 ± 0.10†
RWT/LVM	0.25 ± 0.07	0.14 ± 0.04†
MFS%	57.93 ± 3.35	53.14 ± 7.19†
PWSS (mm/s)	58.71 ± 5.73	40.13 ± 8.79†
Tei index	0.42 ± 0.18	0.45 ± 0.13
TDI-S’ (average mm/s)	5.70 ± 0.79	4.00 ± 0.64†
E/A	1.42 ± 0.11	4.46 ± 2.33†
TDI E’ (average mm/s)	6.44 ± 1.24	5.56 ± 1.34
TDI A’ (average mm/s)	4.79 ± 1.25	4.34 ± 1.9
E/TDI E′	12.6 ± 1.78	21.94 ± 5.32†

Data are mean ± standard deviation. Groups: C (control); AS (Control aortic stenosis). †*p* < 0,05 vs. C. HR, heart rate; LVDD, left ventricular (LV) diastolic diameter; BW, body weight; LA, left atrial diameter; PWT LV, posterior wall thickness; RWT, relative wall thickness; LVM LV, mass; MFS% midwall fractional shortening; PWSS: posterior wall shortening speed; Tei index; TDI S′ tissue Doppler imaging (TDI) of mitral annulus systolic velocity; E/A ratio between early (E)-to-late (A) diastolic mitral inflow. e’, TDI, of early diastolic velocity of mitral annulus; a’, TDI, of end diastolic velocity of mitral annulus; E/e’, E wave to e’ ratio. Student’s t-test or Mann Whitney test.

Echocardiogram performed at 28 weeks demonstrated that AS and AS-Ex groups presented increased diastolic diameter and LV relative thickness, concentric ventricular hypertrophy (LV mass increase), minimized in exercised group. Atrial size (LA/FBW) was lower in AS-Ex animals compared to AS. Functionally, animals in AS-Ex group demonstrated improvement in systolic dysfunction compared to their control, observed increased in MFS and PWSS. Final diastolic dysfunction (increased E/A ratio), RWT/LVM and E/TDI E’ were altered and EX was unable to alleviate this condition in AS group. Significant alterations were observed in TDI-S’, evidencing an improvement in cardiac function parameters of AS-Ex animals compared to AS (Table 2).

**TABLE 2:** Structural and functional echocardiographic data (28 weeks).

Parameters	Groups			
	C (n = 7)	C-EX (n = 7)	AS (n = 8)	AS-EX (n = 8)
HR (bpm)	287 ± 30.67	300 ± 34.07	327 ± 28	311 ± 27
LVDD/BW (mg/kg)	15.33 ± 1.39	14.91 ± 1.59	17.95 ± 1.05*	14.95 ± 1.55#
PWT (mm)	1.66 ± 0.13	1.66 ± 0.13	2.96 ± 0.20*	2.88 ± 0.21
LA/BW (mg/Kg)	9.18 ± 0.99	9.62 ± 1.50	19.50 ± 1.02*	17.18 ± 2.55 #
LVM (g/kg)	1.06 ± 0.24	0.94 ± 0.10	2.24 ± 0.19*	2.18 ± 0.37
RWT (mm)	0.40 ± 0.03	0.43 ± 0.02	0.77 ± 0.09*	0.64 ± 0.10#
RWT/LVM	0.21 ± 0.05	0.23 ± 0.01	0.15 ± 0.03*	0.12 ± 0.02
MFS %	55.78 ± 3.10	57.15 ± 5.86	46.91 ± 4.70*	53.75 ± 3.79#
PWSS (mm/s)	58.57 ± 4.76	65.00 ± 8.02†	39.63 ± 6.14*	57.13 ± 4.29 #
Tei index	0.40 ± 0.16	0.31 ± 0.09	0.40 ± 0.25	0.19 ± 0.07
TDI S’ (average mm/s)	5.61 ± 0.40	5.94 ± 0.36	3.67 ± 0.31*	4.41 ± 0.23#
E/A	1.41 ± 0.26	1.52 ± 0.09	5.08 ± 1.98*	4.92 ± 2.41
TDI E’ (average mm/s)	6.64 ± 0.92	6.47 ± 0.73	6.15 ± 1.23	6.03 ± 1.26
TDI A’ (average mm/s)	4.5 ± 0.58	4.8 ± 1.6	5.1 ± 2.11	3.7 ± 1.33
E/TDI E′	11.46 ± 1.24	13.91 ± 2.5	21.21 ± 7.2*	24.71 ± 4.3

Data are mean ± standard deviation. Groups: C (control); AS (control aortic stenosis); C-Ex (control physical exercise); AS-Ex (aortic stenosis physical exercise). *p < 0.05 vs. C; #*p* < 0.05 vs. AS. HR: heart rate; LVDD: left ventricular (LV) diastolic diameter; BW: body weight; LA: left atrial diameter; PWT: LV, posterior wall thickness; RWT: relative wall thickness; LVM: LV, mass; MFS%: mid-wall fractional shortening; PWSS: posterior wall shortening speed; Tei index; TDI S′ tissue Doppler imaging (TDI) of mitral annulus systolic velocity; E/A ratio between early (E)-to-late (A) diastolic mitral inflow. TDI E′, TDI, of early diastolic velocity of mitral annulus; TDI A′, TDI, of end diastolic velocity of mitral annulus; E/E′, E wave to TDI E′ ratio. ANOVA, two way with Tukey *post hoc*.

### 3.4 Oxidative stress evaluation

**FIGURE 4:**
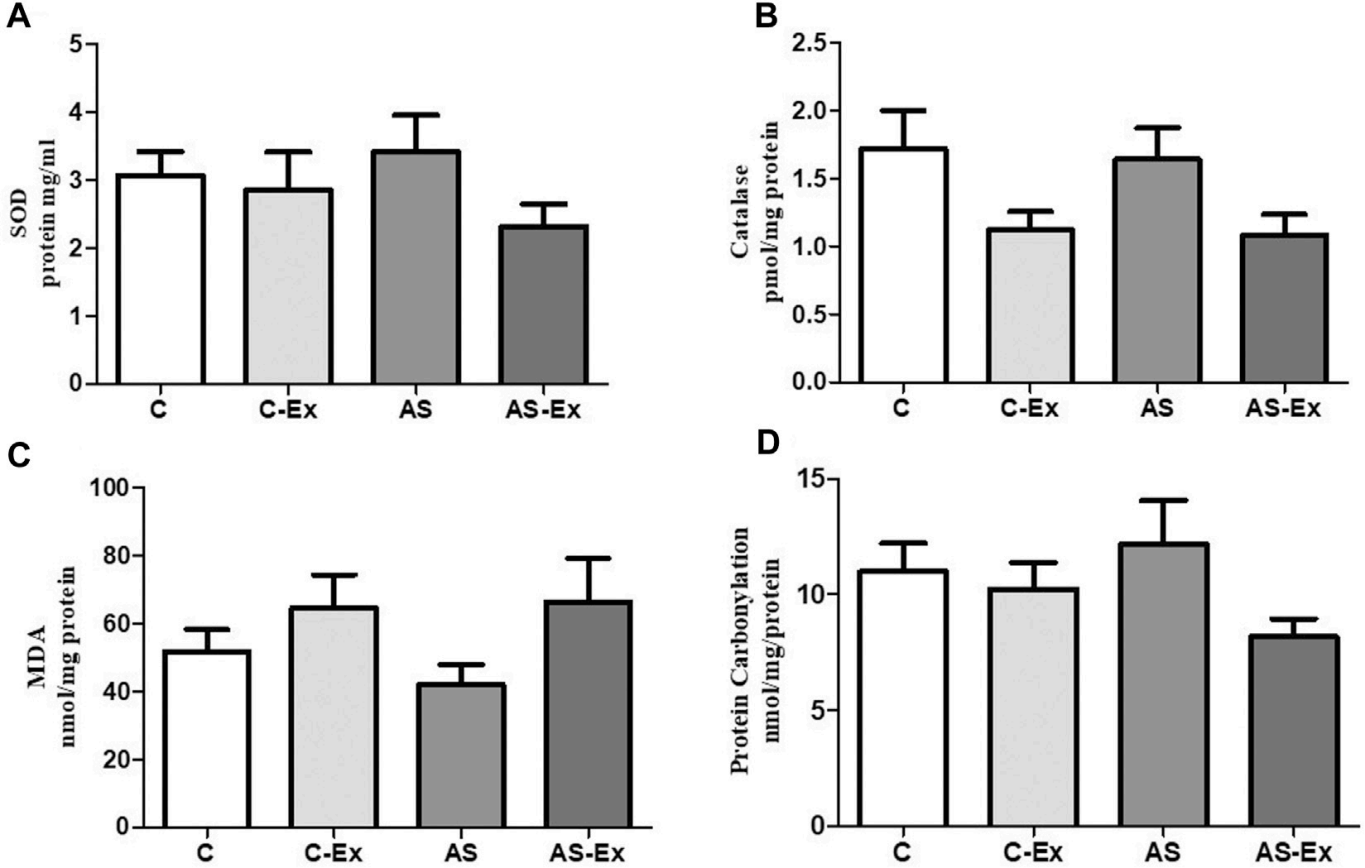
Evaluation of Superoxide Dismutase - SOD **(A)**, Catalase **(B)**, Lipid Peroxidation - MDA **(C)**, and Protein Carbonylation **(D)**. Data expressed as mean ± standard deviation. Groups: C (control, n = 8); C-Ex (control physical exercise, n = 8); AS (control aortic stenosis, n = 8) AS-Ex (aortic stenosis physical exercise, n = 8). ANOVA two way with Tukey *post hoc.*

Activity of antioxidant enzymes SOD and Catalase, and oxidative stress markers did not differ between groups (Figure 4).

### 3.5 Gene expression evaluation: Real-time PCR-RT

**FIGURE 5:**
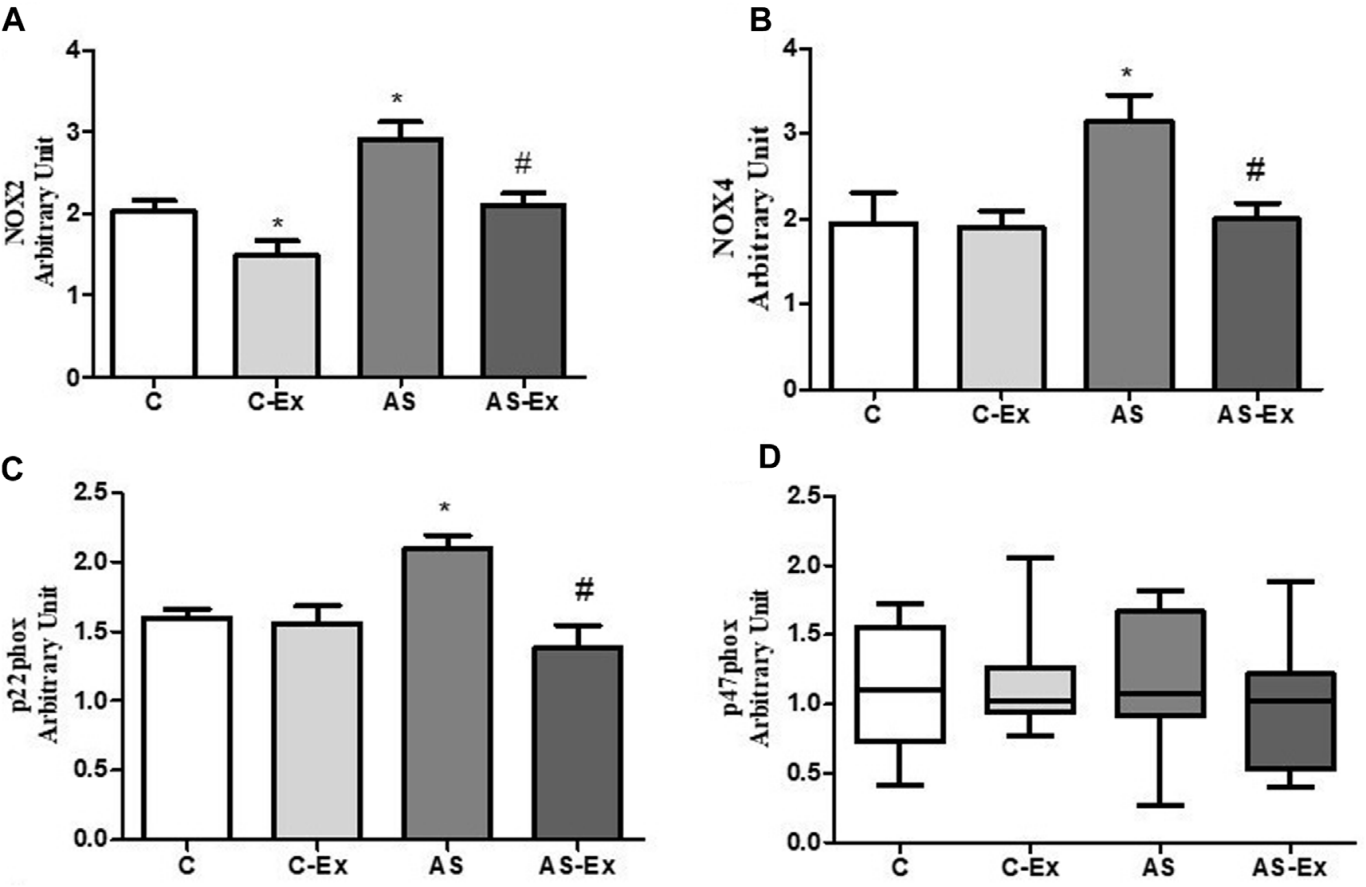
Gene expression of NADPH oxidase subunits. **(A)** Nox2, **(B)** Nox4, **(C)** p22phox, and **(D)** p47phox. Data expressed as mean ± standard deviation or median and percentiles. Groups: C (control, n = 8); C-Ex (control physical exercise, n = 8); AS (control aortic stenosis, n = 8) AS-Ex (aortic stenosis physical exercise, n = 8). p < 0.05. *p < 0,05 vs. C; #*p* < 0,05 vs. AS. ANOVA two way with Tukey *post hoc* or Kruskal-Wallis followed by Dunn’s test.

Aortic stenosis groups showed increased gene expression of Nox2 compared to their respective control. Aerobic exercise protocol was able to reduce Nox2 gene expression in rats with aortic stenosis. As NOX2 activation is dependent of the regulatory subunit p47phox, we also evaluated p47phox gene expression, which did not differ between groups. Aortic stenosis increased Nox4 gene expression compared to control group, which was reduced by aerobic exercise. Aerobic exercise training also reduced the gene expression of the subunit p22phox, required for both NOX2 and NOX4 activation, in aortic stenosis animals. (Figure 5).

### 3.6 Protein expression evaluation

**FIGURE 6:**
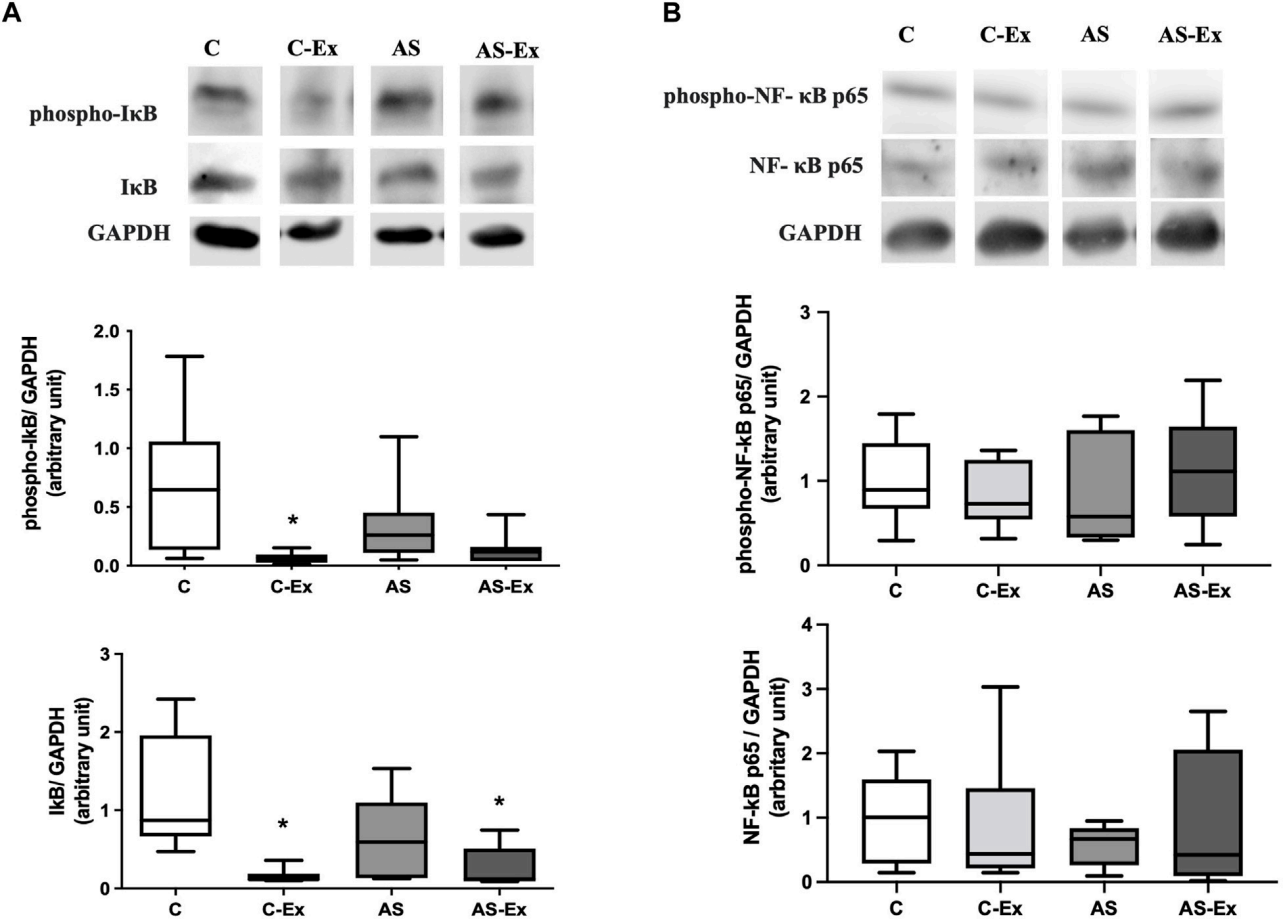
Protein expression of **(A)** inhibitor of nuclear factor kappa B (IκB) and **(B)** nuclear factor kappa B (NF-κB) p65 analyzed by Western blotting. Protein levels were normalized to GAPDH level. Data expressed as median and percentiles. Groups: C (control, n = 7); C-Ex (control physical exercise, *n* = 7); AS (control aortic stenosis, *n* = 7) AS-Ex (aortic stenosis physical exercise, *n* = 7). *p < 0,05 vs. C; Kruskal-Wallis followed by Dunn’s test.

The results of protein expression of NF-κB p65 and its inhibitor IκB are expressed in Figure 6. Phospho-I;A-B expression was lower in C-Ex and AS-Ex groups compared to the C. Total Iκ-B protein expression was lower in C-Ex compared to C group (Figure 6A). Protein expression of NF-κB p65 did not differ between groups (Figure 6B).

Groups: C (control, n = 7); C-Ex (control physical exercise, n = 7); AS (control aortic stenosis, n = 7) AS-Ex (aortic stenosis physical exercise, n = 7). p < 0.05 *. ANOVA two way with Tukey *post hoc*.

## 4 Discussion

**FIGURE 7:**
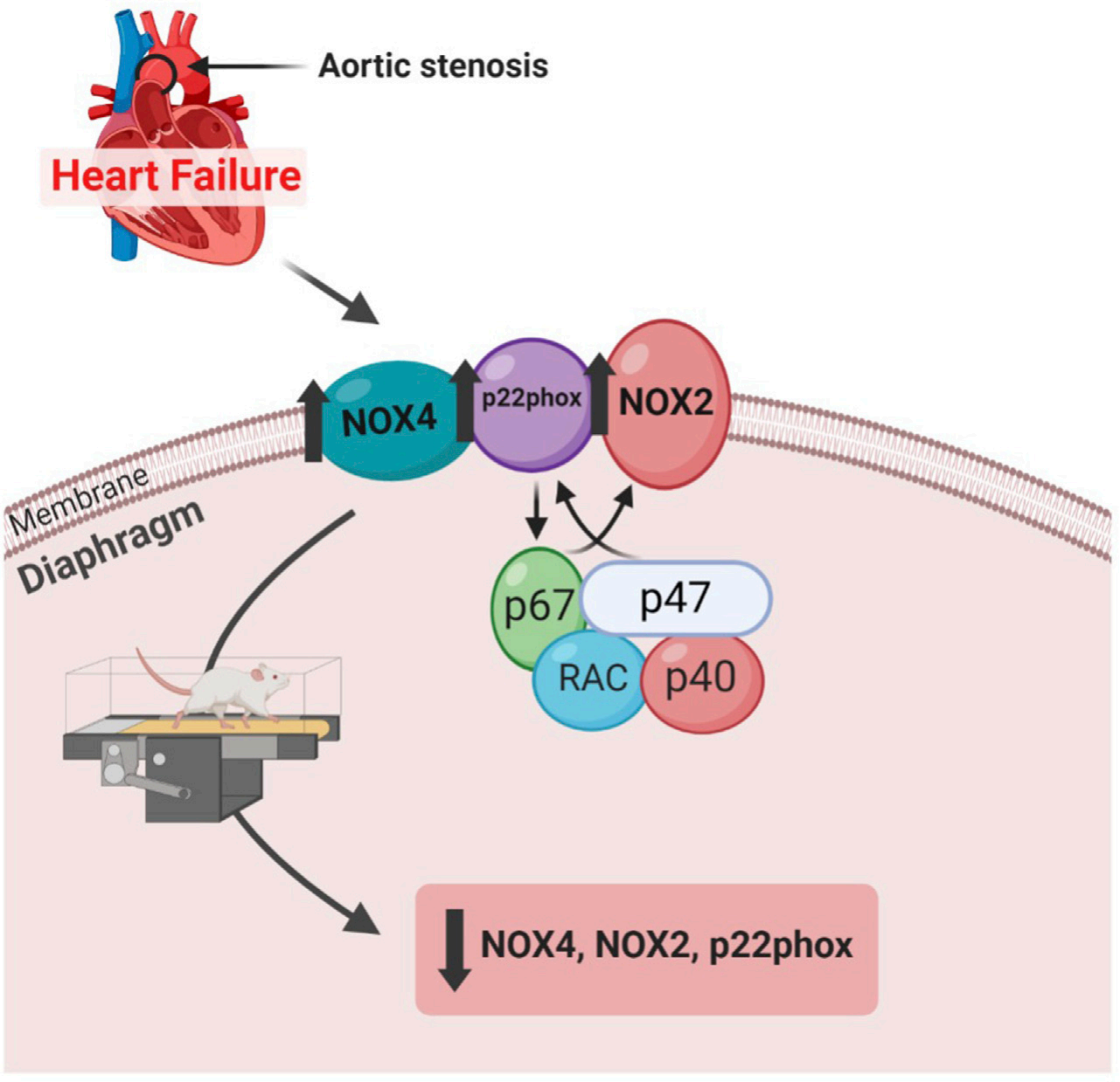
Diagram demonstrating the study findings.

In this study, we demonstrated that aerobic exercise improved cardiac function and decreased gene expression of NADPH oxidase, a major source of reactive oxygen species (ROS), in the diaphragm of rats with aortic stenosis-induced heart failure (Figure 7).

Diaphragmatic dysfunction in HF is related to a predisposition to pneumonia, inability to maintain ventilation during exercise and shallow breathing that impairs alveolar ventilation, that directly affects quality of life, contributing to increased morbidity and mortality due to HF (Van Hees et al., 2010). Diaphragm is the main inspiratory muscle and its myopathy in HF occurs due to its contractile, metabolic properties and responses to stressors (Powers et al., 2013). Previous studies showed that the diaphragm is more sensitive to atrophy than hindlimb muscles and presents alterations in larger magnitudes in HF; mainly due to its oxidative metabolism (Van Hees et al., 2010; Mangner et al., 2013; Bowen et al., 2015).

Experimental aortic stenosis in rats is often used to induce heart failure. This model leads to gradual LV obstruction, LV dysfunction, and later HF (between 18–28 weeks after surgery), similarly to HF development in humans (Mangner et al., 2016). We performed an echocardiogram evaluation at 18 weeks after aortic stenosis surgery to assess the degree of cardiac injury prior to aerobic exercise training. Before exercise, animals with aortic stenosis presented LV and atrial hypertrophy with mild systolic dysfunction and diastolic dysfunction compared to control animals. At the end of the experiment, animals with AS maintained the echocardiographic alterations observed in the initial exam, but with greater severity. However, systolic dysfunction was attenuated in AS animals subjected to aerobic exercise (AS-Ex), characterized by higher values of PWSS, MSF and TDI-S’ compared to AS animals that remained sedentary during the experimental period. Moreover, aerobic exercise minimized diastolic dysfunction in AS animals as well, characterized by an improvement in E/A wave. Our data demonstrated that aerobic exercise is effective to improve cardiac function of rats with HF.

It is well established that cardiac function plays an important role in the development of heart failure, but it is insufficient by its own to explain the progression of the disease (Okita et al., 2013). Skeletal muscle intrinsic abnormalities are associated with the pathogenesis of the symptoms, poor prognosis, and higher mortality in patients with HF (Kinugawa et al., 2015). Interestingly, other studies have demonstrated an increase in functional capacity regardless improvement in echocardiographic parameters in rats with aortic stenosis subjected to an aerobic exercise protocol, which highlighted both respiratory and peripheral musculature as potential effectors of the beneficial effects of exercise (Okita et al., 2013; Mangner et al., 2016).

Skeletal muscle generates significant amounts of reactive oxygen species (ROS), which in physiological levels act as signaling molecules that modulate important biological processes. Exercise results in an acute increase in the production of ROS as evidenced by elevated biomarkers of oxidative damage in both blood and skeletal muscle (Kumar et al., 2023). Zhao et al. demonstrated the effects of exercise programs on the expression of both oxidative and antioxidant markers in skeletal muscle with different time points (Zhao et al., 2013). These acute physiological changes during endurance exercise also plays an important role in cell signaling pathways involved in muscle adaptation to exercise (Kumar et al., 2023). However, sustained high levels of ROS can cause oxidative damage to RNA, DNA, lipids, proteins, and cause cell death (Gomes et al., 2017).

Oxidative stress is defined as an imbalance between the production of ROS and the antioxidant defenses. It has been shown that oxidative stress is increased in heart failure due to both increased generation of ROS and impairment of antioxidant system (Powers et al., 2020). Therefore, we performed an oxidative stress analysis in the diaphragm muscle. We first evaluated muscle concentration of lipid and protein oxidative damage markers, malondialdehyde (MDA) and protein carbonylation, respectively. We, then, determined the activity of the antioxidant enzymes superoxide dismutase and catalase. Oxidative damage markers and antioxidant enzymes activity did not differ between groups.

To determine whether heart failure and aerobic exercise had any influence at the transcriptional level, we evaluated the gene expression of two NADPH oxidase homologs (Nox2 and Nox4) and their regulatory subunits p22phox and p47phox. Although we did not see any change in redox balance, our results revealed altered gene expression of NADPH oxidase in the diaphragm of rats with aortic stenosis, which was protect by aerobic exercise.

The only known function of NADPH oxidases is to produce free radicals. Skeletal muscles express three of the NADPH oxidase isoforms - NOX1, NOX2, and NOX4. NOX 2 and NOX4 have been demonstrated to contribute to heart failure-induced skeletal muscle abnormalities (Ferreira and Laitano, 2016). The role of NADPH oxidase in diaphragm muscle oxidative stress during heart failure is still unclear. In this study, the major component of both NOX2 and NOX4, p22phox, did not differ between the groups, except by lower expression in AS-Ex group compared to AS group. The cytosolic subunit p47phox, which is required for NOX2 activation (Ahn et al., 2017; Reyes et al., 2019) was similar between the experimental groups. However, our data demonstrated that heart failure increased the gene expression of both Nox2 and Nox4 homologs, which was reduced by aerobic exercise. Our results therefore show that aerobic exercise prevented oxidative stress at a transcriptional level. Previous investigations from our research group did not show alterations of gene expression or activity of NADPH oxidases on cardiac (Reyes et al., 2019) and limb skeletal muscles (Gomes et al., 2020) in rats with heart failure, suggesting that perhaps the diaphragm muscle presents alterations in larger magnitudes than other muscles during HF.

Although this study was limited to a transcriptional analysis of NADPH oxidases in the diaphragm muscle of rats with heart failure, previous and recent evidence have demonstrated the impact of this important source of oxidants in skeletal muscle contractile properties (Ahn et al., 2015; Reyes et al., 2019; Powers et al., 2020). A recent study demonstrated that skeletal muscle specific knockout for Nox4 provided full protection against the loss of diaphragm maximal force, while the knockout of Nox2 provided partial protection in mice with heart failure (Kumar et al., 2023).

Exercise is the most effective intervention to prevent or treat skeletal muscle myopathy associated with heart failure (Gomes et al., 2017). It has been demonstrated that increased activity of NADPH in the diaphragm muscle of hypertensive rats led to an oxidant-mediated diaphragm dysfunction, which was reversed by a high intensity interval training (Bowen et al., 2017). On the other hand, it has also been demonstrated that aerobic exercise protocol reduces skeletal muscle oxidative stress, regardless any change in NADPH oxidase activity in rats with aortic stenosis (Mangner et al., 2016; Powers et al., 2020). Differences between experimental models of heart failure, muscle evaluated, or different exercise types and intensities may be responsible for divergent results.

Despite the extensive research interest and growing understanding on exercise for the treatment of several chronic diseases, our current knowledge of the mechanisms by which exercise improves skeletal muscle health is insufficient. Nuclear factor-kappa B (NF-κB) is one of the most important signaling pathways linked to oxidative stress and to the loss of skeletal muscle in several pathological conditions (Zandi et al., 1997; Li et al., 2008). It has been shown an increase in Iκ-B (inhibitor of nuclear factor kappa B) phosphorylation and a decrease in protein levels of p65 NF-κB in soleus muscle of rats with HF (Martinez et al., 2016). Iκ-B phosphorylation occurs via the ubiquitination pathway, where Iκ-B disengages from NF-κB, and the NF-κB migrates to the nucleus, where it binds its target genes (Ghosh and Karin, 2002). In skeletal muscle, NF-κB pathway is linked to inflammation and loss of muscle mass, and cachexia (Bonaldo and Sandri, 2013). In this study, neither aortic stenosis nor exercise modulated the protein levels of Iκ-B in diaphragm muscle of rats with heart failure. Similarly, a previous study have demonstrated positive effect of exercise on oxidative stress, regardless changing on NF-κB pathway (Mangner et al., 2016).

In conclusion, our data revealed that aerobic exercise improves cardiac function in rats with aortic stenosis-induced heart failure. In the diaphragm muscle, aerobic exercise decreases gene expression of NADPH oxidase subunits, demonstrating to be a potential nonpharmacological therapy against oxidative stress in heart failure. It is worth noting that this study evaluated only the diaphragm muscle. It is therefore tempting to explore in future studies whether these exciting findings in respiratory muscle are also observed in muscles with different metabolic and contractile properties, such as peripheral skeletal muscles.

## Data Availability

The original contributions presented in the study are included in the article/supplementary materials, further inquiries can be directed to the corresponding author.

## References

[B1] Ahn B. Beharry A. W. Frye G. S. Judge A. R. Ferreira L. F. (2015). NAD(P)H oxidase subunit p47phox is elevated, and p47phox knockout prevents diaphragm contractile dysfunction in heart failure. Am. J. Physiol. Lung Cell Mol. Physiol. 309 (5), L497–L505. 10.1152/ajplung.00176.2015 26209274 PMC4556931

[B2] Ahn B. Coblentz P. D. Beharry A. W. Patel N. Judge A. R. Moylan J. S. (2017). Diaphragm abnormalities in patients with end-stage heart failure: NADPH oxidase upregulation and protein oxidation. Front. Physiol. 7, 686. 10.3389/fphys.2016.00686 28119629 PMC5220111

[B3] Bechara L. R. G. Moreira J. B. N. Jannig P. R. Voltarelli V. A. Dourado P. M. Vasconcelos A. R. (2014). NADPH oxidase hyperactivity induces plantaris atrophy in heart failure rats. Int. J. Cardiol. 175 (3), 499–507. 10.1016/j.ijcard.2014.06.046 25023789

[B4] Bonaldo P. Sandri M. (2013). Cellular and molecular mechanisms of muscle atrophy. Dis. Model Mech. 6 (1), 25–39. 10.1242/dmm.010389 23268536 PMC3529336

[B5] Bowen T. S. Eisenkolb S. Drobner J. Fischer T. Werner S. Linke A. (2017). High-intensity interval training prevents oxidant-mediated diaphragm muscle weakness in hypertensive mice. FASEB J. 31 (1), 60–71. 10.1096/fj.201600672R 27650398

[B6] Bowen T. S. Rolim N. P. Fischer T. Baekkerud F. H. Medeiros A. Werner S. (2015). Heart failure with preserved ejection fraction induces molecular, mitochondrial, histological, and functional alterations in rat respiratory and limb skeletal muscle. Eur. J. Heart Fail 17 (3), 263–272. Epub 2015 Feb 6. PMID: 25655080. 10.1002/ejhf.239 25655080

[B7] Carvalho R. F. Castan E. P. Coelho C. A. Lopes F. S. Almeida F. L. A. Michelin A. (2010). Heart failure increases atrogin-1 and MuRF1 gene expression in skeletal muscle with fiber type-specific atrophy. J. Mol. Histol. 41, 81–87. 10.1007/s10735-010-9262-x 20349269

[B8] de Souza P. A. T. de Souza R. W. A. Soares L. C. Piedade W. P. Campos D. H. S. Carvalho R. F. (2015). Aerobic training attenuates nicotinic acethylcholine receptor changes in the diaphragm muscle during heart failure. Histol. Histopathol. 30 (7), 801–811. 10.14670/HH-11-581 25548098

[B9] Del Buono M. G. Arena R. Borlaug B. A. Carbone S. Canada J. M. Kirkman D. L. (2019). Exercise intolerance in patients with heart failure: JACC state-of-the-art review. J. Am. Coll. Cardiol. 73 (17), 2209–2225. 10.1016/j.jacc.2019.01.072 31047010

[B10] dos Santos K. C. Cury S. S. Ferraz A. P. C. R. Corrente J. E. Gonçalves B. M. de Araújo Machado L. H. (2018). Recovery of cardiac remodeling and dysmetabolism by pancreatic islet injury improvement in diabetic rats after yacon leaf extract treatment. Oxid. Med. Cell Longev. 2018, 1821359–9. 10.1155/2018/1821359 30057670 PMC6051012

[B11] Ferreira L. F. Laitano O. (2016). Regulation of NADPH oxidases in skeletal muscle. Free Radic. Biol. Med. 98, 18–28. Epub 2016 May 13. PMID: 27184955; PMCID: PMC4975970. 10.1016/j.freeradbiomed.2016.05.011 27184955 PMC4975970

[B12] Ghosh S. Karin M. (2002). Missing pieces in the NF-kappaB puzzle. Cell 109 (1), S81–S96. 10.1016/s0092-8674(02)00703-1 11983155

[B13] Gomes M. J. Martinez P. F. Campos D. H. S. Pagan L. U. Bonomo C. Lima A. R. R. (2016). Beneficial effects of physical exercise on functional capacity and skeletal muscle oxidative stress in rats with aortic stenosis-induced heart failure. Oxid. Med. Cell Longev. 2016, 8695716. 10.1155/2016/8695716 26904168 PMC4745811

[B14] Gomes M. J. Martinez P. F. Pagan L. U. Damatto R. L. Cezar M. D. M. Lima A. R. R. (2017). Skeletal muscle aging: Influence of oxidative stress and physical exercise. Oncotarget 8 (12), 20428–20440. 10.18632/oncotarget.14670 28099900 PMC5386774

[B15] Gomes M. J. Pagan L. U. Lima A. R. R. Reyes D. A. R. Martinez P. F. Damatto F. C. (2020). Effects of aerobic and resistance exercise on cardiac remodelling and skeletal muscle oxidative stress of infarcted rats. J. Cell Mol. Med. 24 (9), 5352–5362. 10.1111/jcmm.15191 32239667 PMC7205792

[B16] Heidenreich P. A. Bozkurt B. Aguilar D. Allen L. A. Byun J. J. Colvin M. M. (2022). 2022 AHA/ACC/HFSA guideline for the management of heart failure: A report of the American college of cardiology/American heart association joint committee on clinical practice guidelines. Circulation 145 (18), e895–e1032. 10.1161/CIR.0000000000001063 35363499

[B17] Jaenisch R. B. Stefani G. P. Durante C. Chechi C. Hentschke V. S. Rossato D. D. (2018). Respiratory muscle training decreases diaphragm DNA damage in rats with heart failure. Can. J. Physiol. Pharmacol. 96 (3), 221–226. Epub 2017 Aug 8. PMID: 28787581. 10.1139/cjpp-2017-0069 28787581

[B18] Kilkenny C. Browne W. J. Cuthill I. C. Emerson M. Altman D. G. (2010). Improving bioscience research reporting: The ARRIVE guidelines for reporting animal research. PLoS Biol. 8 (6), e1000412. 10.1371/journal.pbio.1000412 20613859 PMC2893951

[B19] Kinugawa S. Takada S. Matsushima S. Okita K. Tsutsui H. (2015). Skeletal muscle abnormalities in heart failure. Int. Heart J. 56 (5), 475–484. Epub 2015 Sep 4. PMID: 26346520. 10.1536/ihj.15-108 26346520

[B20] Kumar R. A. Hahn D. Kelley R. C. Muscato D. R. Shamoun A. Curbelo-Bermudez N. (2023). Skeletal muscle Nox4 knockout prevents and Nox2 knockout blunts loss of maximal diaphragm force in mice with heart failure with reduced ejection fraction. Free Radic. Biol. Med. 194, 23–32. 10.1016/j.freeradbiomed.2022.11.025 36436728 PMC10191720

[B21] Li H. Malhotra S. Kumar A. (2008). Nuclear factor-kappa B signaling in skeletal muscle atrophy. J. Mol. Med. Berl. 86 (10), 1113–1126. 10.1007/s00109-008-0373-8 18574572 PMC2597184

[B22] Lima-Leopoldo A. P. Leopoldo A. S. Silva D. C. T. Nascimento A. F. d. Campos D. H. S. d. Luvizotto R. d. A. M. (2013). Influence of long-term obesity on myocardial gene expression. Arq. Bras. Cardiol. 100 (3), 229–237. 10.5935/abc.20130045 23598576

[B23] Mangner N. Bowen T. S. Werner S. Fischer T. Kullnick Y. Oberbach A. (2016). Exercise training prevents diaphragm contractile dysfunction in heart failure. Med. Sci. Sport Exerc 48 (11), 2118–2124. 10.1249/MSS.0000000000001016 27327028

[B24] Mangner N. Linke A. Oberbach A. (2013). Exercise training prevents TNF-α induced loss of force in the diaphragm of mice. Seebacher F. Ed. PLoS One 8 (1), e52274. 10.1371/journal.pone.0052274 PMC3534708 23300968

[B25] Martinez P. F. Bonomo C. Guizoni D. M. Junior S. A. O. Damatto R. L. Cezar M. D. M. (2016). Modulation of MAPK and NF-954;B signaling pathways by antioxidant therapy in skeletal muscle of heart failure rats. Cell Physiol. Biochem. 39 (1), 371–384. 10.1159/000445631 27351177

[B26] Martinez P. F. Okoshi K. Zornoff L. A. M. Carvalho R. F. Oliveira Junior S. A. Lima A. R. R. (2010). Chronic heart failure-induced skeletal muscle atrophy, necrosis, and changes in myogenic regulatory factors. Med. Sci. Monit. 16 (12), BR374–83. 21119570

[B27] Okita K. Kinugawa S. Tsutsui H. (2013). Exercise intolerance in chronic heart failure--skeletal muscle dysfunction and potential therapies. Circ. J. 77 (2), 293–300. Epub 2013 Jan 19. PMID: 23337207. 10.1253/circj.cj-12-1235 23337207

[B28] Pacagnelli F. L. de Almeida Sabela A. K. D. Okoshi K. Mariano T. B. Campos D. H. S. Carvalho R. F. (2016). Preventive aerobic training exerts a cardioprotective effect on rats treated with monocrotaline. Int. J. Exp. Pathol. 97 (3), 238–247. 10.1111/iep.12166 27365256 PMC4960574

[B29] Pagan L. U. Damatto R. L. Cezar M. D. M. Lima A. R. R. Bonomo C. Campos D. H. S. (2015). Long-Term low intensity physical exercise attenuates heart failure development in aging spontaneously hypertensive rats. Cell Physiol. Biochem. 36 (1), 61–74. 10.1159/000374053 25924734

[B30] Powers S. K. Deminice R. Ozdemir M. Yoshihara T. Bomkamp M. P. Hyatt H. (2020). Exercise-induced oxidative stress: Friend or foe? J. Sport Health Sci. 9 (5), 415–425. 10.1016/j.jshs.2020.04.001 32380253 PMC7498668

[B31] Powers S. K. Wiggs M. P. Sollanek K. J. Smuder A. J. (2013). Ventilator-induced diaphragm dysfunction: Cause and effect. Am. J. Physiol. Integr. Comp. Physiol. 305 (5), R464–R477. 10.1152/ajpregu.00231.2013 23842681

[B32] Reyes D. R. A. Gomes M. J. Rosa C. M. Pagan L. U. Zanati S. G. Damatto R. L. (2019). Exercise during transition from compensated left ventricular hypertrophy to heart failure in aortic stenosis rats. J. Cell Mol. Med. 23 (2), 1235–1245. 10.1111/jcmm.14025 30456799 PMC6349163

[B33] Rosa C. M. Gimenes R. Campos D. H. S. Guirado G. N. Fernandes A. A. H. (2016). Apocynin influence on oxidative stress and cardiac remodeling of spontaneously hypertensive rats with diabetes mellitus. Cardiovasc Diabetol. 15 (1), 126. 10.1186/s12933-016-0442-1 27585437 PMC5009715

[B34] Salah H. M. Goldberg L. R. Molinger J. Felker G. M. Applefeld W. Rassaf T. (2022). Diaphragmatic function in cardiovascular disease: JACC review topic of the week. J. Am. Coll. Cardiol. 80 (17), 1647–1659. 10.1016/j.jacc.2022.08.760 36265961

[B35] Samarghandian S. Farkhondeh T. Samini F. Borji A. (2016). Protective effects of carvacrol against oxidative stress induced by chronic stress in rat’s brain, liver, and kidney. Biochem. Res. Int. 2016, 2645237–7. 10.1155/2016/2645237 26904286 PMC4745576

[B36] The National Academies Collection (2011). Guide for the care and use of laboratory animals. Washington, D.C.: National Academies Press. 10.17226/12910

[B37] Uchiyama M. Mihara M. (1978). Determination of malonaldehyde precursor in tissues by thiobarbituric acid test. Anal. Biochem. 86, 271–278. 10.1016/0003-2697(78)90342-1 655387

[B38] Van Hees H. W. Ottenheijm C. A. Granzier H. L. Dekhuijzen P. N. Heunks L. M. (2010). Heart failure decreases passive tension generation of rat diaphragm fibers. Int. J. Cardiol. 141 (3), 275–283. 10.1016/j.ijcard.2008.12.042 19150150

[B39] Van Hees H. W. van der Heijden H. F. Hafmans T. Ennen L. Heunks L. M. Verheugt F. W. (2008). Impaired isotonic contractility and structural abnormalities in the diaphragm of congestive heart failure rats. Int. J. Cardiol. 128 (3), 326–335. 10.1016/j.ijcard.2007.06.080 17689734

[B40] Zandi E. Rothwarf D. M. Delhase M. Hayakawa M. Karin M. (1997). The IkappaB kinase complex (IKK) contains two kinase subunits, IKKalpha and IKKbeta, necessary for IkappaB phosphorylation and NF-kappaB activation. Cell 91 (2), 243–252. 10.1016/s0092-8674(00)80406-7 9346241

[B41] Zhao H. Liu J. Pan S. Sun Y. Li Q. Li F. (2013). SOD mRNA and MDA expression in rectus femoris muscle of rats with different eccentric exercise programs and time points. PLoS One 8 (9), e73634. 10.1371/journal.pone.0073634 24058480 PMC3772806

